# Targeted Development of Registries of Biological Parts

**DOI:** 10.1371/journal.pone.0002671

**Published:** 2008-07-16

**Authors:** Jean Peccoud, Megan F. Blauvelt, Yizhi Cai, Kristal L. Cooper, Oswald Crasta, Emily C. DeLalla, Clive Evans, Otto Folkerts, Blair M. Lyons, Shrinivasrao P. Mane, Rebecca Shelton, Matthew A. Sweede, Sally A. Waldon

**Affiliations:** Virginia Bioinformatics Institute, Blacksburg, Virginia, United States of America; Tata Institute of Fundamental Research, India

## Abstract

**Background:**

The design and construction of novel biological systems by combining basic building blocks represents a dominant paradigm in synthetic biology. Creating and maintaining a database of these building blocks is a way to streamline the fabrication of complex constructs. The Registry of Standard Biological Parts (Registry) is the most advanced implementation of this idea.

**Methods/Principal Findings:**

By analyzing inclusion relationships between the sequences of the Registry entries, we build a network that can be related to the Registry abstraction hierarchy. The distribution of entry reuse and complexity was extracted from this network. The collection of clones associated with the database entries was also analyzed. The plasmid inserts were amplified and sequenced. The sequences of 162 inserts could be confirmed experimentally but unexpected discrepancies have also been identified.

**Conclusions/Significance:**

Organizational guidelines are proposed to help design and manage this new type of scientific resources. In particular, it appears necessary to compare the cost of ensuring the integrity of database entries and associated biological samples with their value to the users. The initial strategy that permits including any combination of parts irrespective of its potential value leads to an exponential and economically unsustainable growth that may be detrimental to the quality and long-term value of the resource to its users.

## Introduction

D*e novo* gene synthesis [1]–[3] is catalyzing a transition from the *ad-hoc* methods of traditional genetic engineering to the development of industrial-scale fabrication processes enabling users to quickly obtain from commercial vendors genetic constructs that would have been assembled through a custom cloning strategy just a few years ago. Designing a construct for gene synthesis often consists in combining a number of previously defined DNA sequences [4]. The design of an expression cassette in *Escherichia coli* typically includes a promoter, a Ribosome Binding Site (RBS), a coding sequence, and a transcription terminator. These functional blocks are commonly referred to as biological parts or genetic parts. Catalogues of biological parts that are sufficiently well characterized to be used in the design of new genetic constructs can be described in review articles [5], embedded into software applications to design new DNA sequences [4], or made available through a web site [6], [7]. With four years of existence and 4,856 entries in July 2007, the Registry is the largest publicly available library of genetic parts. The Registry goes beyond just cataloguing parts. The parts in the Registry must meet the BioBrick standard, which requires the part sequence to be framed by standard cloning sites called the prefix and suffix. If the part sequences do not contain any of the restriction sequences used by the prefix and suffix, this standardization ensures that it is possible to use a generic cloning process to combine two BioBrick-compliant parts. The process is generic because the restriction enzymes and ligation steps it includes are independent of the sequences of the two parts being combined. This standardized assembly of new genetic constructs derived from standardized parts is therefore complementary to *de novo* gene synthesis since both approaches can be used to fabricate designer DNA sequences. Another benefit from standardizing parts is the physical composition of BioBrick parts. The restriction sites used by the BioBrick standard ensure that the combination of two BioBrick parts results in a new BioBrick part that can be added to the list of parts available for future design projects. The composition of parts leads to distinguishing two categories of parts. Composite parts are parts resulting from the composition of two parts whereas basic parts are parts that cannot be decomposed into smaller parts.

In addition to developing a large catalogue of parts, the Registry has developed a repository of 995 bacterial clones (as of July 2007) corresponding to physical implementations of entries in the Registry database. The Registry database content and clone collection have been primarily developed by students enrolled in the in the International Genetically Engineered Machine (iGEM) competition [8], [9]. Each year, the iGEM organizers send the entire clone collection to all the teams enrolled in the competition. The teams use this toolkit to implement the designs required for their project. At the end of the summer, the teams contribute back to the Registry new basic parts and new composite parts they have made during the course of their project. This new material is included in the Registry and becomes available to the teams enrolled in the competition the following year. If students enrolled in iGEM still represent the largest group of Registry users, recent publications have demonstrated that this resource can enable the development of more mainstream research projects [10]–[14].

We have analyzed the Registry to identify usage patterns that could help design the next generation of infrastructures to host libraries of genetic parts. The analysis consists of two parts. First, the structure of the database itself is considered in terms of the relationships between database entries by examining their published sequences and categorization. The Registry uses two levels of categories to organize its content. Entries of different functional types (promoters, coding sequences, etc.) are regrouped into three classes according to their level of complexity. The simplest entries are found at the bottom of the hierarchy in a class labeled “Parts”. Combinations of parts implementing specific functions like inversion of a signal, gene expression cassettes, or reporter genes are found in “Devices”. Finally self-contained combinations of devices designed for a particular application are placed under “Systems” [7]. This categorization implements an abstraction hierarchy, an approach commonly used in engineering to manage complex engineering projects by allowing different groups of specialists to work at different levels. Ultimately, engineers with a domain expertise should be able to develop application-specific systems by combining previously characterized devices without having to know more about these devices than their operational characteristics. The second part of our analysis is a comparative analysis of the published sequences of database entries and the experimental sequences of the corresponding clones, which we obtained by sequencing the clones in one distribution of the DNA repository.

While a library of parts as a single centralized community resource has clear benefits, there are still many reasons for organizations or individual investigators to structure their own libraries of parts [15], [16]. These reasons may include the physical or legal availability of a limited set of parts, previous experience with a specific parts list, the use of specific organisms not included in community resources, the inclusion of proprietary parts in the design, and possibly others. Hence, our results have implications beyond the analysis of a specific resource at a particular point in time.

## Results

### Analysis of the database content

Since most Registry entries correspond to constructs that have not yet been fabricated, it appeared more interesting to limit the analysis of the database to the 995 entries for which a clone was available. Among these 995 entries, 279 were in the “Parts” layer of the Registry abstraction hierarchy. The remaining 716 entries were categorized in the “devices” and “systems” layers of the hierarchy. In this paper we use parts to refer to entries in the “parts” layer and design for entries categorized in the device or systems layers of the hierarchy.

**Figure 1:**
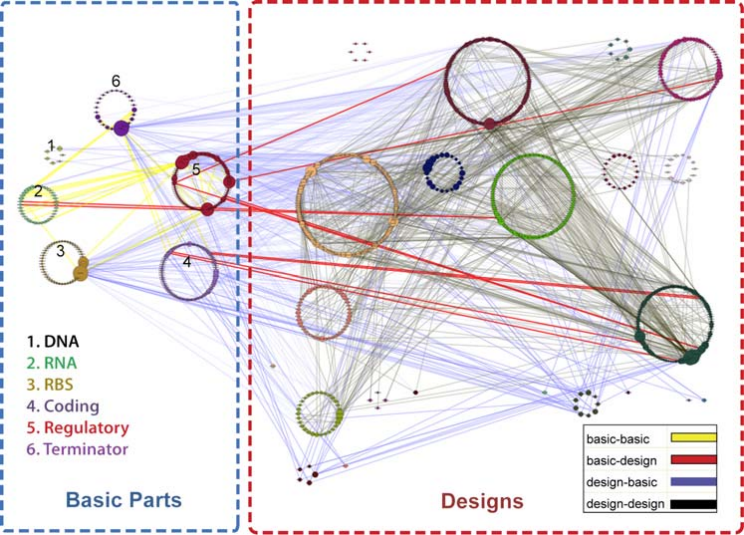
Network of inclusion relationships among the Registry entries. Nodes of this network correspond to entries in the Registry. Nodes are grouped in color-coded circles according to the Registry categories. Categories corresponding to parts are within the blue box on the left side of the figure whereas categories corresponding to designs are located within the red box on the right side. The diameter of the nodes corresponds to the node connectivity. The directed edges indicate that the sequence of one entry is included in the sequence of another entry. Edges are color-coded according to the type of relationship. If most of the edges correspond to natural relations (parts included in designs, and designs included in other designs), it is somewhat surprising that parts can include other parts (yellow edges) and it is unclear why some parts would include design in their sequence (red edges). Detailed analysis of individual entries can be conducted using a Cytoscape [28] file (Figure S1).

We derived a network of relationships between entries in the Registry from their published sequence. First, inclusion relationships between entries were identified by pair wise comparison of the sequences in the database. Entry A is connected to entry B if the sequence of A includes the sequence of B. In a second step, this directed graph was pruned to eliminate transitive relationships. For instance, if A includes B and B includes C, then a relationship between A and C can be derived from the previous relationships. In this example the inclusion of C within A is pruned from the graph. This operation allowed us to draw a network of 1383 relationships among the 995 entries considered in this analysis (Figure 1 and Figure S1). We identified 496 relationships in which the sequences of designs included part sequences. We also found 826 inclusion relationships between design sequences. Since parts correspond to the bottom layer of the abstraction hierarchy, it was expected that there would be few if any connections among entries in this group. However, 49 relationships between parts have also been identified. Even more surprising, 12 relationships indicated that entries in the design group were present in the sequences of parts. These observations appear to be inconsistent with the Registry abstraction hierarchy.

After having identified inclusion relationships within the Registry, we summarized this pruned connection matrix by computing for each entry, the number of other entries directly included in its sequence (a measure of its design complexity) and the number of entries in which its sequence is found (a measure of its popularity). The joint distribution of entry complexity and popularity provides a global perspective on the dynamics of design reuse to build more complex designs (Table 1). Entries in the first column (299 entries) are true basic parts while the entries that have never been reused are in the first line (502 entries). If some entries have been used in as many as 70 designs (Table 2), 80% have been used less than 3 times. Because indirect relationships have been removed from the pruned interaction network, the complexity axis on Table 1 does not refer to the total number of parts included in the design but it indicates the number of subcomponents an entry is composed of. Approximately 50% of the entries can be broken down into two other entries, which is consistent with a pair wise assembly process. It also indicates that users have recorded most of the construction intermediates. The ideal shape of this joint distribution is not clear except that few entries should be located near the origin. The value of having a lot of parts used very infrequently is questionable, so the weight of the popularity distribution should shift away from 0.

**Table 1:** Joint-distribution of the parts complexity and popularity.

Popularity^1^	Complexity^2^							
	*0*	*1*	*2*	*3*	*4*	*5*	*6*	*Sum*
*0*	154	64	236	39	5	4	-	502
*1*	65	24	150	12	1	1	2	255
*2*	30	9	62	10	3	-	-	114
*3*	21	7	19	1	-	-	-	48
*4*	8	3	7	-	-	-	-	18
*5*	6	4	5	-	-	-	-	15
*6*	3	-	7	-	-	-	-	10
*7*	3	-	3	1	-	-	-	7
*8*	-	-	1	-	-	-	-	1
*9*	2	-	3	-	-	-	-	5
*10*	1	-	1	-	-	-	-	2
*11*	2	-	1	-	-	-	-	3
*12*	1	-	-	-	-	-	-	1
*13*	-	-	-	-	-	-	-	0
*14*	-	1	1	-	-	-	-	2
*15*	-	-	-	-	-	-	-	0
*16*	-	-	3	-	-	-	-	3
*17*	-	1	-	-	-	-	-	1
*18*	-	-	-	-	-	-	-	0
*19*	-	-	1	-	-	-	-	1
*20*	1	-	-	-	-	-	-	1
*21*	-	-	-	-	-	-	-	0
*22*	1	-	-	-	-	-	-	1
*…*	-	-	-	-	-	-	-	0
*31*	-	-	1	-	-	-	-	1
*…*	-	-	-	-	-	-	-	0
*36*	-	-	1	-	-	-	-	1
*…*	-	-	-	-	-	-	-	0
*39*	-	-	1	-	-	-	-	1
*…*	-	-	-	-	-	-	-	0
*52*	1	-	-	-	-	-	-	1
*…*	-	-	-	-	-	-	-	0
*70*	-	-	1	-	-	-	-	1
*Sum*	299	113	504	63	9	5	2	995

1 Number of times Registry entries are used in other entries

2 Number of entries included in an entry sequence

**Table 2:** The Registry most popular parts.

ID	Category	N^1^	Description	Parts included
BBa_B0015	Terminator	70	Double terminator consisting of BBa_B0010 and BBa_B0012	BBa_B0010, BBa_B0012
BBa_B0034	RBS	52	RBS based on Elowitz repressilator	-
BBa_E0430	Reporter	39	Standard YFP Output Device -LVA tag	BBa_E0130, BBa_S01014
BBa_E0432	Reporter	36	EYFP (RBS+ LVA+ TERM) (B0034.E0032.B0015)	BBa_I9045, BBa_S01638
BBa_J13002	Regulatory	31	TetR repressed POPS/RIPS generator	BBa_B0034, BBa_R0040
BBa_R0040	Regulatory	22	TetR repressible promoter	-
BBa_R0011	Regulatory	20	Promoter (lacI regulated, lambda pL hybrid)	-
BBa_I0500	Regulatory	19	Inducible pBad/araC	BBa_I13458, BBa_R0080
BBa_B0030	RBS	17	Strong RBS based on Ron Weiss thesis	BBa_B0034
BBa_I13507	Composite	16	Screening plasmid intermediate	BBa_I13501, BBa_I13502
BBa_I13504	Reporter	16	Screening plasmid intermediate	BBa_I13401, BBa_I13500
BBa_S03155	Intermediate	16	Trminators B0010+B0012+promoter R0040	BBa_B0015, BBa_R0040
BBa_J04500	Intermediate	14	IPTG inducible promoter with RBS	BBa_B0034, BBa_R0010
BBa_Q04121	Inverter	14	LacI QPI with strong RBS, hybrid promoter	BBa_P0412
BBa_R0062	Regulatory	12	Promoter activated by LuxR in concert with HSL	-
BBa_E0420	Reporter	11	Standard CFP output device w/o LVA tag	BBa_B0015, BBa_S01022
BBa_R0051	Regulatory	11	promoter (lambda cI regulated)	-
BBa_B0032	RBS	11	Weak1 RBS based on Ron Weiss thesis	-
BBa_Q04400	Inverter	10	TetR QPI with strong RBS	BBa_P0440, BBa_S03155
BBa_B0031	RBS	10	RBS.2 (weak) – derivative of BBa_0030	-

1 Entry popularity.

### Analysis of the DNA repository

The analysis of the Registry database reveals some of the challenges in implementing the abstraction hierarchy upon which this community resource has been built. However, making the parts physically available adds another level of complexity. We have therefore systematically analyzed the library of plasmids shipped in May 2007 to teams enrolled in the iGEM competition.

The plasmids were distributed lyophilized in four 384-well plates. After suspending the DNA into water, the solutions were quantified using a spectrophotometer and only two wells did not appear to contain any DNA. In order to obtain enough material for DNA sequencing, the inserts were amplified using primers complementary to the standardized prefix and suffix used to clone them into the vector. The products of amplification were analyzed by electrophoresis to select clones suitable for sequencing. In particular, we eliminated 216 clones that did not amplify and 190 clones that resulted in multiple peaks of size greater than 120 bp. The lack of amplification product can either result from a problem with the amplification reaction or indicate the absence of sequences complementary to the primers. The presence of multiple peaks may be caused by primer dimers, non-specific amplification, or the presence of different plasmids in the well. Since parts with sequences shorter than 120 bp can easily be obtained as oligonucleotides, only the 789 clones that generated a single PCR fragment larger than 120 bp were sequenced. Of these 789 sequenced clones, 509 have published sequences that were used for subsequent analyses.

**Figure 2:**
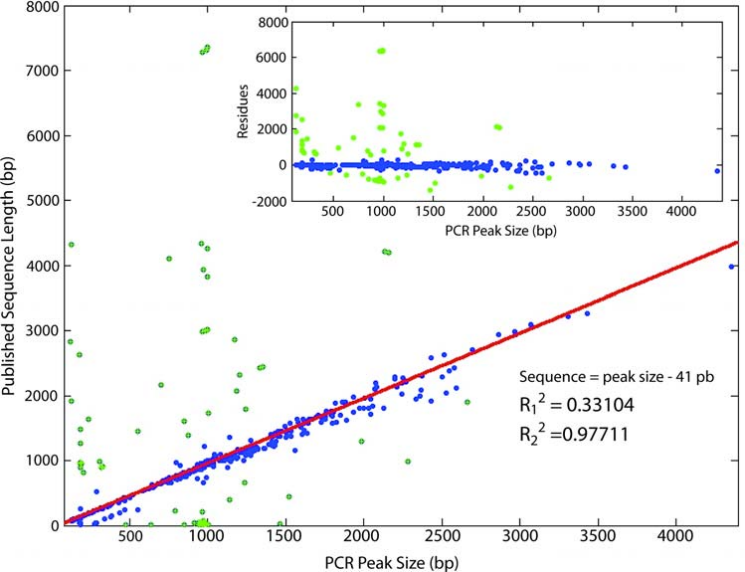
Comparison of the Registry published sequences with the size of the PCR amplification products. This plot is limited to the clones that generated a single PCR fragment greater than 120 bp. Theoretically, the size of the PCR fragment is 41 pb longer than the length of the published sequence because of the presence of the PCR primer sequences in the amplification product (n = 509). When all data points were used in the linear regression, the fit led to a coefficient of correlation R_1_^2^ = 0.33. Based on previously reported experimental error affecting fragment size determination[17], 76 outliers were eliminated manually (green points) leading to a greatly improved R_2_^2^ = 0.98.

To get a global measure of the match between published and physical sequences, we plotted the length of the published sequence against the size of the PCR fragment for the 509 sequenced plasmids having a documented sequence. On Figure 2, 76 outliers were visually identified. The rest of the lengths remained close to the expected lengths. Yet, only 285 data points had less than a 10% difference between the two sequence lengths. The differences in size distribution between measured and expected lengths appears wider than 5%, the previously reported experimental error affecting the determination of fragment size by the microfluidic system used for this project [17]. We have not investigated all discrepancies, amplification failures, or multiplicity of amplification products. This would require a systematic curation of the published sequences, as well as individual PCR troubleshooting for each clone, which were beyond the scope of this project.

The next step of our analysis was to look at sequences individually. Of the 789 clones sequenced, 591 could be assembled in contigs. The length of assembled sequences ranges from 166 bp to 1897 bp. Some of the inserts that could not be assembled may be too long to achieve a significant overlap between the two sequence files starting from both extremities of the insert. Assembling these clones would require additional sequencing runs utilizing clone-specific primers. Out of the 591 clones assembled, only 354 could be associated with a Registry entry as the other clones were undocumented in the particular distribution of the Registry used in this project. The assembled sequences were aligned with the published sequence using BLAST [18]. Out of 354 assembled sequences for which published sequences were available, 334 produced alignments with their published sequence and the complete results of this alignment analysis are reported in Table S1. This spreadsheet was used to identify clones for which the assembled sequence confirms the published sequence. Since the assembled sequence can include the primer sequences, the assembled sequence should not be longer than the published sequence plus the combined length of the two primers (41 bp). Since the primers used in this project are adjacent to the sequence being verified, the first and last 10 to 25 bases of the insert can be difficult to read. As a result, the assembled sequence may be up to 50 bp shorter than the published sequence. These two criteria led to the selection of 221 clones for which −41≤length (published sequence) – length(assembled sequence) ≤50. In the second step of sequence analysis, we want to ensure that the alignment of the assembled and published sequences covers most of the shorter of the two sequences. In this second step, from the 221 clones meeting the assembled sequence length criteria, we selected 177 clones for which the alignment length is at least 99% of the length of the smaller of the two sequences being compared. After these two rounds of selections, the percentage of identity of the assembled and published sequence was always superior or equal to 97% and greater or equal to 99% for 162 of the 177 clones. It is obvious that different choices of parameters would lead to larger or smaller number of clones with a confirmed published sequence.

Just like in the case of PCR results, a systematic control of the published sequences could improve the clone confirmation statistics. It is quite possible that for a number of these clones the biological material is correct but their published sequence may be inaccurate. Additional sequencing runs starting from within the insert sequences would also increase the number of clones with long inserts that could be confirmed.

## Discussion

### A global analysis of the Registry

This analysis of the DNA library provides no more than a snapshot of one distribution of the Registry clone collection. Amplification and sequencing problems could result from technical problems during the experiments described in this paper just as they could indicate problems with the biological samples themselves. For instance, samples that lead to multiple amplification products could have been delivered contaminated, could have been contaminated during one of the steps described in this report, or could simply result from mispriming. In order to control the experiments, it would be necessary to repeat all the operations starting from a new series of samples. Unfortunately the lack of unique clone identifier makes such control problematic. The different distributions of the DNA repository do not share a common key necessary to relate one distribution to another. The data set described in this article is specific to the 2007 distribution of the DNA repository. Our results are valuable to understand global issues associated with the design, development, and management of a registry of biological parts but they would need data describing how different distributions relate to each other to be used for controlling the quality of specific clones in the Registry collection.

The high-level analysis of the Registry database led to the identification of several non-trivial issues that need to be addressed. The implementation of a workable abstraction hierarchy remains problematic. A single category of parts (DNA) appears to be exclusively composed of basic building blocks. However, our sequence analysis has revealed elements categorized as parts within the Registry that include other parts, indicating that not all clones categorized as parts have an atomic nature. Some part sequences even include designs, a higher level in the abstraction hierarchy. These observations result from the lack of consensus in the community on how biological parts should be defined. Nothing illustrates this confusion better than the complex architecture of promoters [19], [20]. On the one hand, promoters are generally considered as parts but on the other hand they have well characterized domains that can be associated with specific functions. When developing an abstraction hierarchy, should promoters lie at its bottom and be considered as atomic parts or should they be considered as composite parts composed of multiple functional domains? The case of genes is not simpler as proteins are also composed of multiple functional domains [21]. A complete access to the Registry database would have made it possible to investigate questions that could not be addressed using the partial dump of the database content used in this analysis. For instance, parts have a usefulness attribute used to report if a part works, works with issues, or does not work as anticipated. It would be interesting to relate the parts popularity to the usefulness status of a part as one would imagine that the most popular parts are reported as working. The structure of composite parts is also described as the series of basic parts they are composed of. Comparing the sequence and structure of composite parts could help investigate a number of interesting questions. Figure 1 reflects the laborious efforts of the synthetic biology community to develop and implement the new theoretical framework it needs to support its scientific vision.

### Organizational guidelines

Results presented in this report lead to a number of organizational guidelines that could help design or manage registries of biological parts.

The published DNA sequence of entries should be carefully curated. Lack of published sequences or incorrect ones hamper the quality control of the associated clones. It is important to clearly identify basic parts of a registry as they generate the rest of the database. Basic parts should be linked and compared to entries in other sequence databases and peer-reviewed publications [5]. Basic parts that have not been completely annotated should be flagged so that people considering using them may proceed with caution. The sequence redundancy of the basic part set is a difficult problem. Theoretically, a set of basic parts could be atomic in the sense that it generates all other entries in a registry. However, this approach may not always be practical. If certain projects need to identify several parts in a promoter sequence, this level of granularity may be excessive for other projects. The same argument can apply at higher levels of organization. For the same reasons, nothing prevents the definition of complete gene expression cassettes and other devices such as switches, inverters, etc. as basic parts. However, this option does not seem desirable as it would be inconsistent with the engineering vision of building complex systems from a limited numbers of building blocks.

The integrity of the sequence of composite parts is even more difficult to ensure. There could either be a static or dynamic link between the sequence of a composite part and the sequences of the basic parts it is composed of. In the first case, the sequence of a composite part is automatically derived from the sequence of its components when the composite part is created but future changes to the sequences of its basic components do not propagate to the composite part sequence. If such a policy is enforced, discrepancies between the composite part sequence and the sequences of its basic components can develop over time. It is desirable that such discrepancies be identified. In the case of a dynamic link, any change in the sequence of a basic part propagates to all composite parts using this basic part. The integrity of the composite part sequence is then always preserved but different versions of the composite parts that are automatically generated by this process may be very confusing to the users.

Clones in the DNA repository associated to a parts registry need to be uniquely identified independently of the parts in the registry. Parts numbers are not good identifiers of clones as many clones correspond to the same part in different plasmids or different bacterial strains. A clone key is necessary to compare data collected on different distributions of the same clone and therefore implement quality control procedures. A standardized quality control process should be specified to ensure the integrity of the clone collection.

### Targeted development of registries of parts

The idea of developing collections of standardized parts is a transformative idea in biology [22]. After a few years of a large scale experiment, it becomes apparent that developing and managing this new type of resource for synthetic biology raises a number of original questions. Specialized registries built on compatible standards are being developed by various groups that will experiment with different user interfaces, workflows, and modes of user interaction. These initiatives along with future developments of the original Registry will provide elements of solutions to these new questions.

It will be particularly interesting to see if different registries will adopt different editorial policies. The cost of maintaining a parts registry depends on its size as each entry needs to be properly documented and each clone needs to be verified. Parts registries are different from traditional collections used in biological research as any combination of parts in the registry can also be integrated in the registry. A small number of basic parts can therefore generate a potentially infinite collection of clones. Initially, it may be attractive to record any combination of parts without any consideration of its potential value, but this approach now appears unsustainable [16]. At some point, users and managers of a parts registry will need to analyze the allocation of their resources. Table 2 and similar analysis on other registries can help identify entries that are the most valuable to the users and least expensive to maintain. For instance, basic parts deserve special attention because they enable the development of new designs and errors affecting basic parts can propagate to the entire resource. This contrasts with the case of a large and specialized construct including multiple genes that would be expensive to control and might have a low probability of reuse. Even though it would be desirable to also include such construct in the database and clone collection, if finite resources require choosing between recording a few new basic parts with a broad reuse potential and a specialized and expensive part, it is likely that resources will be preferably allocated to adding basic parts. Similarly, including a switch that could be used in developing a number of applications will probably be deemed more valuable than the construction intermediates that were generated during its assembly. Managers of parts registries need to articulate editorial policies to set criteria for including new entries in their database so that resources can be targeted to developing content maximizing the benefits to their users.

Recognizing that repositories of biological parts are an essential component of the upcoming integrated development environments for synthetic biology [6], [23], [24] may help target the development of their content. In order to support this integration it is necessary to specify a minimal data model allowing programmatic access to the registry databases from multiple client applications. A draft of such a data model is described in Text S1. Structured methods for designing synthetic genetic systems will provide a theoretical framework that will guide the development of user interfaces helping users combine basic parts into complex designs. Alternative solutions to the organization of parts in categories or the mechanism to define composite parts will probably be proposed. In this context, recent initiatives to organize forums aiming at defining technical standards for biological parts appear very timely and laudable.

## Materials and Methods

The plasmids were resuspended in 30 µl of nuclease free water (Ambion) at 4°C overnight. They were quantified using the Nanodrop spectrophotometer. 20 ng of Plasmid DNA was used in the PCR amplification of the plasmid inserts, using Qiagen's Taq PCR master mix kit, and 2 µM primers forward and reverse primers at 100 µl reaction volume. The forward primer was homologous to the BioBrick prefix (5′ - GAA TTC GCG GCC GCT TCT AG - 3′) whereas the reverse primer was complementary to the suffix sequence (5′ - CTG CAG CGG CCG CTA CTA GTA - 3′). PCR conditions: 94°C 45 sec, (94°C 30 sec, 55°C 45 sec, 72°C 45 sec) for 24 cycles, 72°C 5 minutes, 4°C hold.

The PCR product was purified using Qiagen's QIAquick PCR purification kit, resuspended in 25 µl of nuclease free water, and quality controlled using the Agilent Bioanalyser DNA 7500 assay. The amplified products were quantified and diluted to 10 ng per µl. The PCR product and corresponding primers were submitted to the VBI Core Laboratory for Sanger sequencing using the primers used in the amplification step. Sequencing conditions: 400 ng template DNA, 3.2 pmol primer, 2.5 ul BigDye Terminator mix v3.1, water to a total volume 15 µl.

Base calling and quality control of sequence chromatograms was done by PHRED [25], [26]. The sequences were assembled using CAP3 [27] with default options except for minimum overlap size of 21 bp. The assembled sequences were aligned with their respective published sequences using BLAST [18] with default parameters.

## Supporting Information

Figure S1 Cytoscape file used to generate Figure 1. Can be used to interactively explore the network of relationships within the Registry
(0.10 MB ZIP)

Table S1 Blast analysis of the clones assembled sequences against the published sequence.
(0.14 MB XLS)

Text S1 Describes the supporting database and its data model. Also describes the other files included in the supplement.
(0.12 MB PDF)
