# Supplementary material for: Targeted Development of Registries of Biological Parts
Source: PLoS One. 2008 Jul 16;3(7):e2671. doi: 10.1371/journal.pone.0002671 (PMC2441434; doi:10.1371/journal.pone.0002671)
Supplement: Text S1 — Describes the supporting database and its data model. Also describes the other files included in the supplement. (0.12 MB PDF) [file pone.0002671.s003.pdf]

Targeted development of registries of biological parts – online supplement

Jean Peccoud*, Megan F. Blauvelt, Yizhi Cai, Kristal L. Cooper, Oswald Crasta, Emily DeLalla, Clive Evans, Otto Folkerts, Blair Lyons, Rebecca Shelton, Shrinivasrao P. Mane, Matthew A. Sweede, Sally A. Waldon

***Corresponding author.**

Database infrastructure

A custom MySQL database was developed to organize the data generated by this project. The database can be accessed at www.genocad.org/phpMyAdmin using the parameters below:

login name: biobrick_viewer
password: parts

Below is the description of the tables in this database along with a short description of the fields when needed.

- **dna_repository**: Our database is organized around the notion of clones which are the plasmids distributed to the iGEM teams. This table is therefore the spine of our database.
    - **CloneID**: a unique identifier of the clones.
    - **Bba_number**: this is the key used by the MIT Registry to identify its entries. This key points to the biobrick_parts table.
    - **Plasmid**: This key points to the plasmid table used to record the plasmid backbone in which the parts are inserted.
    - **physical_sequence**: this is the sequence assembled from multiple sequencing runs.
    - **Selected_for_sequencing**: Indicates if the clone was sequenced
    - **Rec_date**: date of last modification of the record.
- **Biobrick_parts:** This table records information relative to biological parts, i.e. functional DNA fragment.
    - **ID:** a unique identifier for the biobricks.
    - **Bba_number:** The identifier used by the Registry of Standard Biological Parts
    - **Dna_sequence:** the part theoretical or published sequence. This sequence information was imported from the Registry.
    - **Part_type:** An integer used to indicate the category of the parts. Categories are listed in the part_type table. They correspond to the

functional categories used by the Registry such as Ribosome Binding Site, Regulatory, Coding, etc.

  - **description**: A short description of the part.
  - **Rec_date**: date of last modification of the record.

- **Plate_key**: The DNA Repository came in 4 384-well plates that we remapped to a set of 16 96-well plates. For each clone in the repository, this table indicates in which well it can be found in the 384-well and 96-well sets.
- **Peaks**: This table is used to record the electrophoresis data after the PCR step. Each clone is associated through the cloneID field to 0 or more DNA fragments or electrophoresis peaks in this table. The fields describing the peaks are documented in the user manual of the Agilent 2100 Expert software.
- **Nanodrop**: This table is used to record the spectrophotometric data generated by the software controlling the Nanodrop instrument.
- **Parts_contained_sequences**: This table is used to record the sequence inclusion relationships used to draw Figure 1. The master_sequence_id is the identifier of a BioBrick whose sequence contains the contained_sequence_id. Master_part_type and contained_sequence_type points to the category of each of the corresponding parts. The fields corresponding to start and end positions of the alignment are self-explanatory. Relationship_flagged is used to indicated the reduced set of relationships. Relations flagged with 1 constitute the reduced set used to generate Figure 1.

Downloadable files

The following supporting files are available in the online supplement:

- `Registry.cys` is the files used to generate figure 1. By importing this file into Cytoscape, it is possible to interactively explore the Registry connectivity diagram.
- `MyVBIonBBa_tophits.xls` is an Excel file summarizing the results of the Blast analysis that can be used to analyze the quality of the match between published and assembled sequence on a clone by clone basis.

In addition, trace files can be downloaded from www.genocad.org/biobricks .`Trace_files.zip` is an archive containing the trace files generated by the sequencers. They can be used to reassemble the sequences using different parameters. This archive also contains sequence data for clones that could not be assembled.

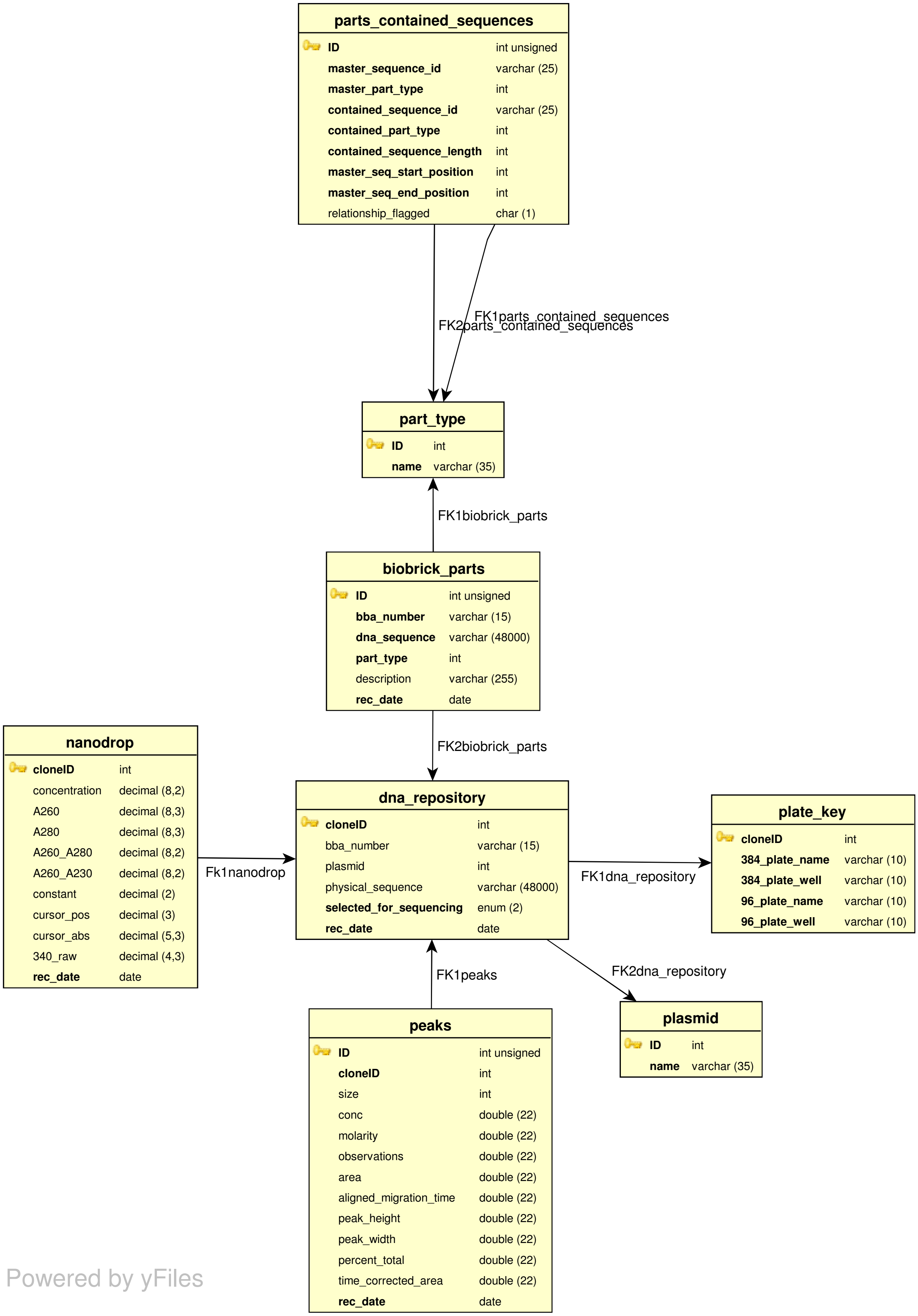

Powered by yFiles
